# Potential interaction between lysophosphatidic acid and tumor-associated macrophages in ovarian carcinoma

**DOI:** 10.1186/s12950-020-00254-4

**Published:** 2020-08-05

**Authors:** Ying Feng, Meizhu Xiao, Zihan Zhang, Ran Cui, Xuan Jiang, Shuzhen Wang, Huimin Bai, Chongdong Liu, Zhenyu Zhang

**Affiliations:** 1 Department of Obstetrics and Gynecology, Beijing Chao-Yang Hospital, Capital Medical University, No. 8, North Road of Workers Stadium, Chaoyang District, Beijing, 100020 China; 2 Department of Gynecology and Obstetrics, Peking Union Medical College Hospital, Chinese Academy of Medical Sciences & Peking Union Medical College, Beijing, China

**Keywords:** Ovarian carcinoma, Tumor microenvironment, Tumor associated macrophage, LPA, LPAR, PI3K/AKT/mTOR, PPAR γ

## Abstract

Ovarian carcinoma is the deadliest type of gynecological cancer. The unique tumor microenvironment enables specific and efficient metastasis, weakens immunological monitoring, and mediates drug resistance. Tumor associated macrophages (TAMs) are a crucial part of the TME and are involved in various aspects of tumor behavior. Lysophosphatidic acid (LPA) is elevated in the blood of ovarian carcinoma patients, as well as in the tumor tissues and ascites, which make it a useful biomarker and a potential therapeutic target. Recent studies have shown that LPA transforms monocytes into macrophages and regulates the formation of macrophages through the AKT/mTOR pathway, and PPAR γ is a major regulator of LPA-derived macrophages. In addition, TAMs synthesize and secrete LPA and express LPA receptor (LPAR) on the surface. With these data in mind, we hypothesize that LPA can convert monocytes directly into TAMs in the microenvironment of ovarian cancer. LPA may mediate TAM formation by activating the PI3K/AKT/mTOR signaling pathway through LPAR on the cell surface, which may also affect the function of PPAR γ, leading to increased LPA production by TAMs. Thus, LPA and TAMs form a vicious circle that affects the malignant behavior of ovarian cancer.

## Introduction

Ovarian carcinoma is the most common cause of mortality from gynecological tumors and the 5th leading cause of cancer death in women [1]. The five-year survival rate is only approximately 46.5% [2]. Several characteristics of ovarian cancer are related to its lethality, including the exfoliation of tumor cells, metastasis and diffusion through peritoneal fluid, and tumor promotion and immunosuppression by the tumor microenvironment (TME) [3]. As an important component of the TME, tumor associated macrophages (TAMs) make a crucial part in ovarian cancer progression, chemotherapeutic resistance, immunosuppression and prognosis. At present, there have been some reports on immunotherapy targeting TAMs [4–7].

### Roles of TAMs in ovarian cancer

The main characteristic of ovarian cancer is early metastasis through peritoneal fluid. Ascites contain a large population of TAMs [8–10], forming a unique microenvironment [11]. Macrophages can inhibit apoptosis, promote tumor invasion and proliferation, suppress antitumor immune cells and foster tumor angiogenesis [12, 13]. TAMs in the ovarian cancer are generated from monocytes and resident macrophages. Research has shown that ovarian cancer TAMs are similar to monocyte-derived macrophages [14], which adopt the M2 phenotype. TAMs promote tumor progression, chemotherapeutic resistance and immunosuppression [11, 12, 15–17]. CD163 and CD206/MRC1, which are strongly expressed on TAMs, are receptors for immunosuppressive molecules and predict the early recurrence of ovarian cancer [18–20]. CD163 and CD206 mRNA expression is also associated with IL-10 levels in ascites, which indicate a shorter relapse free survival (RFS) in patients with ovarian cancer [21]. The prognosis and survival of ovarian cancer patients are related to the presence of TAMs. Several adverse clinical markers are highly expressed by ovarian cancer TAMs, including CD163, Procollagen C-endopeptidase enhancer 2 (PCOLCE2), IL-6 and IL-10 [22]. TAMs are the primary secretors of most collagens and the main source of most protease inhibitors and make an important part in the synthesis of extracellular matrix (ECM) proteins [11]. Macrophages play a crucial role in ECM remodeling and the invasion of ovarian cancer [14, 20, 23]. TAMs can synthesize chemokine ligand 5 (CCL5), chemokine ligand 8 (CXCL8), and selectively synthesize CCL18, CXCL2 and CXCL3, all of which can attract monocytes/macrophages and promote tumor progression [24].

### Roles of LPA in ovarian cancer

Lysophosphatidic acid (LPA) was initially identified as a ovarian cancer growth factor and was known as an ovarian cancer activator [25, 26] and a potential marker of ovarian cancer [27]. LPA can promote ascites formation and tumorigenesis [28]. The raised levels of LPA in blood, tissues and ascites make it a useful biomarker and a potential therapeutic target in ovarian carcinoma [29]. LPA can promote tumor survival and proliferation, cisplatin resistance and increase the production of urokinase plasminogen activator (uPA), additional LPA generation and vascular endothelial growth factor (VEGF) in ovarian cancer. LPA can promote the production of protease and neovascularization mediators, and reduce the apoptosis of tumor cells [30], but has no obvious effect on normal ovarian cells [31]; these roles of LPA are similar to those of TAMs. The tissues and cells in the ovarian carcinoma TME maybe the main source of the increased LPA. The cells involved in LPA production include immune cells, platelets, mesothelial cells and adipocytes [29]. The leading role of LPA in ovarian carcinoma is in cell invasion and migration, and these effects are mainly induced by LPA receptors (LPARs). LPARs are a group of G protein-coupled receptors (GPCRs) for LPA that include LPAR1, LPAR2, LPAR3, LPAR4, LPAR5 and LPAR6 [32–39].. Recent studies showed that LPA is related to the formation of ovarian carcinoma stem cells and enhances their malignant behavior; these effects are mediated by LPAR1 [40, 41], which interacts with CD14 [42], a monocyte differentiation antigen which is highly expressed on the cell membrane surface of monocytes/macrophages.

## The hypothesis

**Fig. 1:**
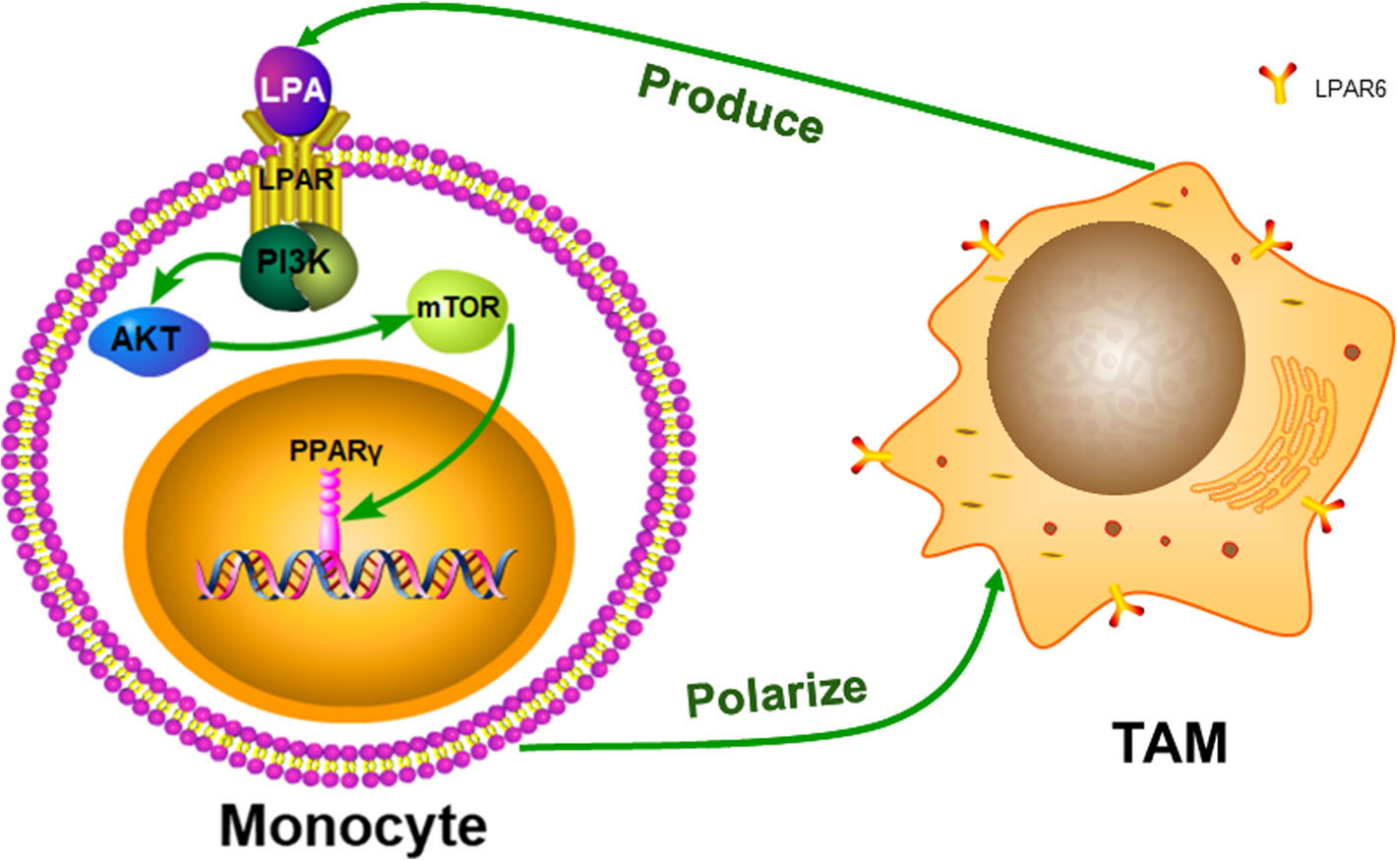
The possible molecular mechanism of LPA induced monocyte polarization to TAM in ovarian cancer TME

The interplay between LPA and tumor associated macrophages plays a critical role in driving ovarian cancer malignancy and offers a potential target for therapy. We propose that this hypothesis is supported by three points, as showed in the Fig. 1:

1. LPA transforms monocytes directly into TAMs in the ovarian cancer TME.
2. LPA regulates TAM formation by activating the PI3K/AKT/mTOR signaling pathway through LPAR on the cell surface, which may also affect the function of peroxisome proliferators-activated receptor gamma (PPAR γ).
3. TAMs produce more LPA. Together, LPA and TAMs form a vicious circle that affects the malignant behavior of ovarian cancer.

## Evaluation of the hypothesis

### LPA and TAMs play similar roles

LPA regulates a variety of tumor-promoting factors and inflammatory factors in epithelial ovarian cancer, including IL-6, IL-8, VEGF, matrix metallopeptidases (MMPs), CXCL12, cytochrome c oxidase subunit 2 (COX2), uPA, cyclin D1, CXCL1 [43]. Macrophages produce many factors that contribute to tumor growth, including VEGF, IL-1, IL-6, nuclear factor-kappa B (NF-κB), tumor necrosis factor-alpha (TNF-α) and macrophage colony-stimulating factor (M-CSF) [44, 45]. LPA enhances the expression and secretion of IL-13 in T cells [46]. M2 macrophages can be activated by IL-13 or IL-4 [47]. The macrophage derived phospholipase PLA2G7 can produce extra-cellular LPA, which participate in the progress of ovarian carcinoma, and is related to the early recurrence of ovarian cancer [21, 48]. LPA helps cancer cells avoid the immune system by improving the chemotaxis of Th2 cells [49] and inhibiting the activation of CD8+ T cells [50]. M2 macrophages have poor antigen-presenting ability, participate in the Th2 reaction, inhibit Th1 adaptive immunity, and promote tumor progression [51, 52].

TAMs express high levels of tumor growth factors and cytokines, such as CCL18, KITLGG, semaphorin-6B, S100 calcium-binding protein B (S100B) and VEGFB. Furthermore, TAMs preferentially express cytokines and growth factors, such as CCL18, that promote tumor progression, growth and recurrence in ovarian cancer [11]. The levels of CCL18 in cancer tissue are related to metastasis and the shorter overall survival of patients with ovarian cancer, which seems to be associated with the increase in the mTOR Complex 2 (mTORC2) signaling pathway [53]. LPARs (LPAR1–6) are GPCRs and the LPA signal is mainly induced by these six GPCRs, which activate extracellular signal-regulated kinases 1/2 (ERK1/2), phosphoinositide 3-kinase (PI3K), mammalian target of rapamycin (mTOR), Ca2+ mobilization, RAC, RAS and Rho, and stimulate ovarian cancer cell survival and migration [54, 55]. Therefore, we conjecture that LPA is involved in the functions of TAMs, perhaps in a manner related to the PI3K/AKT/mTOR signaling pathway (Fig. 1).

### Relationship between TAMs and LPA

The functional annotation of TAM genes in the ovarian cancer TME most frequently reveals the GPCRs pathway. TAMs participate in the formation of lipids and play a crucial role in the synthesis of LPA. Additionally, macrophages and T cells express LPAR5 and LPAR6 [56]. LPAR6 is the main LPA receptor expressed on ovarian cancer TAMs [11]. In ovarian cancer, LPA is mainly generated by TAMs, and the role of LPA in macrophage polarization was previously reported [57]. Furthermore, it is TAMs, not tumor cells, which produce LPA in fat-free medium. LPA-induced genes in macrophages are related to cell movement and migration in ovarian cancer microenvironment [56]. Accordingly, ovarian cancer TAMs may activate LPAR and the relevant signaling pathway by synthesizing and secreting LPA, thus promoting the invasion and metastasis of ovarian cancer (Fig. 1).

LPA regulates macrophage polarization [11]. Recent research has shown that LPA can convert human monocytes into macrophages [57]. LPA-stimulated macrophages express high levels of CD68 and levels of CD14, CD64, CD68 and CD206 comparable to those expressed by macrophages stimulated with human M-CSF. AKT/mTOR signaling stimulated by LPA makes a significant part in the development of murine macrophages, and PPAR γ is an important transcriptional regulator of LPA-induced macrophage development [57]. Existing data prompt the hypothesis that LPA may activate the PI3K/AKT/mTOR pathway through LPAR to directly induce the polarization of monocytes/macrophages to TAMs in ovarian cancer. This mechanism needs to be better understood in future studies before the application of clinical immunotherapy in ovarian cancer (Fig. 1).

## Conclusion

In ovarian carcinoma, elevated levels of LPA in blood, tissue and ascites lead to the conversion of monocytes into ovarian cancer TAMs in the TME. The molecular mechanism may involve LPA binding to LPAR, which activates the PI3K/AKT/mTOR pathway and affects the function of PPAR γ, resulting in a cascade of reactions and changes. Finally, LPA produced by TAMs and TAMs themselves form a vicious circle that affects the metastasis and invasion of ovarian carcinoma. Further study of the interaction between TAMs and LPA in ovarian cancer will bring about a better further understanding of ovarian cancer pathogeny and will provide theoretical evidence for the treatment and early diagnosis of ovarian carcinoma. This vicious circle is a potential target of immunomodulatory therapy for ovarian cancer.

## Data Availability

Not applicable.

## Abbreviations

TAMs: Tumor associated macrophages
LPA: Lysophosphatidic acid
LPAR: Lysophosphatidic acid receptor
TME: Tumor microenvironment
PI3K: Phosphoinositide 3-kinase
mTOR: Mammalian target of rapamycin
RFS: Relapse free survival
VEGF: Vascular endothelial growth factor
NF-κB: Nuclear factor-kappa B
TNF-α: Tumor necrosis factor-alpha
M-CSF: Macrophage colony-stimulating factor
GPCRs: G protein-coupled receptors
PPARγ: Peroxisome proliferators-activated receptor gamma
PCOLCE2: Procollagen C-endopeptidase enhancer 2
CCL: Chemokine (C-C motif) ligand
CXCL: Chemokine (C-X-C motif) ligand
uPA: urokinase plasminogen activator
MMP: Matrix metallopeptidase
COX2: Cytochrome c oxidase subunit 2
S100B: S100 calcium-binding protein B
mTORC2: mTOR Complex 2
ERK: Extracellular signal-regulated kinases

## References

[1] Siegel RL, Miller KD, Jemal A (2018). Cancer statistics, 2018. CA Cancer J Clin.

[2] Noone AM HN, Krapcho M, Miller D, Brest A, Yu M, Ruhl J, Tatalovich Z, Mariotto A, Lewis DR, Chen HS, Feuer EJ, Cronin KA (eds). SEER Cancer Statistics Review (CSR) 1975-2015, based on November 2017 SEER data submission, posted to the SEER web site, April 2018. Bethesda, MD: national Cancer institute. Updated September 10, 2018.

[3] Pogge von Strandmann E, Reinartz S, Wager U, Muller R (2017). Tumor-host cell interactions in ovarian Cancer: pathways to therapy failure. Trends Cancer..

[4] Kumar S, Ramesh A, Kulkarni A (2020). Targeting macrophages: a novel avenue for cancer drug discovery. Expert Opin Drug Discovery.

[5] Pyonteck SM, Akkari L, Schuhmacher AJ, Bowman RL, Sevenich L, Quail DF (2013). CSF-1R inhibition alters macrophage polarization and blocks glioma progression. Nat Med.

[6] Cannarile MA, Weisser M, Jacob W, Jegg AM, Ries CH, Ruttinger D (2017). Colony-stimulating factor 1 receptor (CSF1R) inhibitors in cancer therapy. J Immunotherapy Cancer.

[7] Sankhala KK, Blay J-Y, Ganjoo KN, Italiano A, Hassan AB, Kim TM (2017). A phase I/II dose escalation and expansion study of cabiralizumab (cabira; FPA-008), an anti-CSF1R antibody, in tenosynovial giant cell tumor (TGCT, diffuse pigmented villonodular synovitis D-PVNS). J Clin Oncol..

[8] Leinster DA, Kulbe H, Everitt G, Thompson R, Perretti M, Gavins FN (2012). The peritoneal tumour microenvironment of high-grade serous ovarian cancer. J Pathol.

[9] Negus RP, Stamp GW, Hadley J, Balkwill FR (1997). Quantitative assessment of the leukocyte infiltrate in ovarian cancer and its relationship to the expression of C-C chemokines. Am J Pathol.

[10] Worzfeld T, Pogge von Strandmann E, Huber M, Adhikary T, Wagner U, Reinartz S, et al. The Unique Molecular and Cellular Microenvironment of Ovarian Cancer Frontiers in oncology 2017;7:24. 10.3389/fonc.2017.00024 PMC5319992 28275576

[11] Worzfeld T, Finkernagel F, Reinartz S, Konzer A, Adhikary T, Nist A (2018). Proteotranscriptomics reveal signaling networks in the ovarian Cancer microenvironment. Mol Cell Proteomics.

[12] Qian BZ, Pollard JW (2010). Macrophage diversity enhances tumor progression and metastasis. Cell..

[13] Engblom C, Pfirschke C, Pittet MJ (2016). The role of myeloid cells in cancer therapies. Nat Rev Cancer.

[14] Finkernagel F, Reinartz S, Lieber S, Adhikary T, Wortmann A, Hoffmann N (2016). The transcriptional signature of human ovarian carcinoma macrophages is associated with extracellular matrix reorganization. Oncotarget..

[15] Condeelis J, Pollard JW (2006). Macrophages: obligate partners for tumor cell migration, invasion, and metastasis. Cell..

[16] Gabrilovich DI, Ostrand-Rosenberg S, Bronte V (2012). Coordinated regulation of myeloid cells by tumours. Nat Rev Immunol.

[17] Sica A, Bronte V (2007). Altered macrophage differentiation and immune dysfunction in tumor development. J Clin Invest.

[18] Burt BM, Rodig SJ, Tilleman TR, Elbardissi AW, Bueno R, Sugarbaker DJ (2011). Circulating and tumor-infiltrating myeloid cells predict survival in human pleural mesothelioma. Cancer..

[19] Quatromoni JG, Eruslanov E (2012). Tumor-associated macrophages: function, phenotype, and link to prognosis in human lung cancer. Am J Transl Res.

[20] Reinartz S, Schumann T, Finkernagel F, Wortmann A, Jansen JM, Meissner W (2014). Mixed-polarization phenotype of ascites-associated macrophages in human ovarian carcinoma: correlation of CD163 expression, cytokine levels and early relapse. Int J Cancer.

[21] Reinartz S, Finkernagel F, Adhikary T, Rohnalter V, Schumann T, Schober Y (2016). A transcriptome-based global map of signaling pathways in the ovarian cancer microenvironment associated with clinical outcome. Genome Biol.

[22] Adhikary T, Wortmann A, Finkernagel F, Lieber S, Nist A, Stiewe T, et al. Interferon signaling in ascites-associated macrophages is linked to a favorable clinical outcome in a subgroup of ovarian carcinoma patients 2017;18(1):243. 10.1186/s12864-017-3630-9 PMC5359932 28327095

[23] Burleson KM, Casey RC, Skubitz KM, Pambuccian SE, Oegema TR, Skubitz AP (2004). Ovarian carcinoma ascites spheroids adhere to extracellular matrix components and mesothelial cell monolayers. Gynecol Oncol.

[24] Sokol CL, Luster AD. The chemokine system in innate immunity. Cold Spring Harb Perspect Biol. 2015;7(5):a016303. 10.1101/cshperspect.a016303 PMC4448619 25635046

[25] Xu Y, Gaudette DC, Boynton JD, Frankel A, Fang XJ, Sharma A (1995). Characterization of an ovarian cancer activating factor in ascites from ovarian cancer patients. Clin Cancer Res.

[26] Xu Y, Casey G, Mills GB (1995). Effect of lysophospholipids on signaling in the human Jurkat T cell line. J Cell Physiol.

[27] Xu Y, Shen Z, Wiper DW, Wu M, Morton RE, Elson P (1998). Lysophosphatidic acid as a potential biomarker for ovarian and other gynecologic cancers. Jama..

[28] Li H, Zhao Z, Wei G, Yan L, Wang D, Zhang H (2010). Group VIA phospholipase A2 in both host and tumor cells is involved in ovarian cancer development. FASEB J.

[29] Xu Y. Lysophospholipid Signaling in the Epithelial Ovarian Cancer Tumor Microenvironment. Cancers. 2018;10(7):227. 10.3390/cancers10070227 PMC6071084 29987226

[30] Eder AM, Sasagawa T, Mao M, Aoki J, Mills GB (2000). Constitutive and lysophosphatidic acid (LPA)-induced LPA production: role of phospholipase D and phospholipase A2. Clin Cancer Research.

[31] Fang X, Gaudette D, Furui T, Mao M, Estrella V, Eder A (2000). Lysophospholipid growth factors in the initiation, progression, metastases, and management of ovarian cancer. Ann N Y Acad Sci.

[32] Taniguchi R, Inoue A, Sayama M, Uwamizu A, Yamashita K, Hirata K (2017). Structural insights into ligand recognition by the lysophosphatidic acid receptor LPA6. Nature..

[33] Sengupta S, Xiao YJ, Xu Y (2003). A novel laminin-induced LPA autocrine loop in the migration of ovarian cancer cells. FASEB J.

[34] Sengupta S, Wang Z, Tipps R, Xu Y (2004). Biology of LPA in health and disease. Semin Cell Dev Biol.

[35] Ren J, Xiao YJ, Singh LS, Zhao X, Zhao Z, Feng L (2006). Lysophosphatidic acid is constitutively produced by human peritoneal mesothelial cells and enhances adhesion, migration, and invasion of ovarian cancer cells. Cancer Res.

[36] Cai H, Xu Y (2013). The role of LPA and YAP signaling in long-term migration of human ovarian cancer cells. Cell Communication Signaling.

[37] Kim KS, Sengupta S, Berk M, Kwak YG, Escobar PF, Belinson J (2006). Hypoxia enhances lysophosphatidic acid responsiveness in ovarian cancer cells and lysophosphatidic acid induces ovarian tumor metastasis in vivo. Cancer Res.

[38] Sengupta S, Kim KS, Berk MP, Oates R, Escobar P, Belinson J (2007). Lysophosphatidic acid downregulates tissue inhibitor of metalloproteinases, which are negatively involved in lysophosphatidic acid-induced cell invasion. Oncogene..

[39] Fan Q, Cai Q, Xu Y (2015). FOXM1 is a downstream target of LPA and YAP oncogenic signaling pathways in high grade serous ovarian cancer. Oncotarget..

[40] Fan Q, Cai Q, Li P, Wang W, Wang J, Gerry E (2017). The novel ZIP4 regulation and its role in ovarian cancer. Oncotarget..

[41] Seo EJ, Kwon YW, Jang IH, Kim DK, Lee SI, Choi EJ (2016). Autotaxin regulates maintenance of ovarian Cancer stem cells through Lysophosphatidic acid-mediated Autocrine mechanism. Stem Cells.

[42] Zhao J, He D, Su Y, Berdyshev E, Chun J, Natarajan V (2011). Lysophosphatidic acid receptor 1 modulates lipopolysaccharide-induced inflammation in alveolar epithelial cells and murine lungs. Am J Physiol Lung Cell Molecular Physiol.

[43] So J, Wang FQ, Navari J, Schreher J, Fishman DA (2005). LPA-induced epithelial ovarian cancer (EOC) in vitro invasion and migration are mediated by VEGF receptor-2 (VEGF-R2). Gynecol Oncol.

[44] Lin EY, Li JF, Gnatovskiy L, Deng Y, Zhu L, Grzesik DA (2006). Macrophages regulate the angiogenic switch in a mouse model of breast cancer. Cancer Res.

[45] Stix G (2007). A malignant flame. Understanding chronic inflammation, which contributes to heart disease, Alzheimer's and a variety of other ailments, may be a key to unlocking the mysteries of cancer. Sci Am.

[46] Rubenfeld J, Guo J, Sookrung N, Chen R, Chaicumpa W, Casolaro V (2006). Lysophosphatidic acid enhances interleukin-13 gene expression and promoter activity in T cells. Am J Physiol Lung Cell Molecular Physiol.

[47] Gordon S, Taylor PR (2005). Monocyte and macrophage heterogeneity. Nat Rev Immunol.

[48] Wong JL, Obermajer N, Odunsi K, Edwards RP, Kalinski P (2016). Synergistic COX2 induction by IFNgamma and TNFalpha self-limits Type-1 immunity in the human tumor microenvironment. Cancer Immunol Res.

[49] Wang L, Knudsen E, Jin Y, Gessani S, Maghazachi AA (2004). Lysophospholipids and chemokines activate distinct signal transduction pathways in T helper 1 and T helper 2 cells. Cell Signal.

[50] Benesch MGK, Yang Z, Tang X, Meng G, Brindley DN (2017). Lysophosphatidate signaling: the tumor Microenvironment's new nemesis. Trends Cancer.

[51] Biswas SK, Mantovani A (2010). Macrophage plasticity and interaction with lymphocyte subsets: cancer as a paradigm. Nat Immunol.

[52] Noel W, Raes G, Hassanzadeh Ghassabeh G, De Baetselier P, Beschin A (2004). Alternatively activated macrophages during parasite infections. Trends Parasitol.

[53] Wang Q, Tang Y, Yu H, Yin Q, Li M, Shi L (2016). CCL18 from tumor-cells promotes epithelial ovarian cancer metastasis via mTOR signaling pathway. Mol Carcinog.

[54] Jesionowska A, Cecerska-Heryc E, Matoszka N, Dolegowska B (2015). Lysophosphatidic acid signaling in ovarian cancer. J Recept Signal Transduct Res.

[55] Samadi N, Bekele R, Capatos D, Venkatraman G, Sariahmetoglu M, Brindley DN (2011). Regulation of lysophosphatidate signaling by autotaxin and lipid phosphate phosphatases with respect to tumor progression, angiogenesis, metastasis and chemo-resistance. Biochimie..

[56] Reinartz S, Lieber S, Pesek J, Brandt DT, Asafova A, Finkernagel F (2019). Cell type-selective pathways and clinical associations of lysophosphatidic acid biosynthesis and signaling in the ovarian cancer microenvironment. Mol Oncol.

[57] Ray R, Rai V (2017). Lysophosphatidic acid converts monocytes into macrophages in both mice and humans. Blood..

